# Effects of PVY-Infected Tobacco Plants on the Adaptation of *Myzus persicae* (Hemiptera: Aphididae)

**DOI:** 10.3390/insects13121120

**Published:** 2022-12-05

**Authors:** Yingqin He, Wenbin Jiang, Wei Ding, Wenlong Chen, Degang Zhao

**Affiliations:** 1 College of Tea Science, Guizhou University, Guiyang 550025, China; 2 College of Plant Protection, Southwest University, Chongqing 400715, China; 3 Guizhou Provincial Key Laboratory for Agricultural Pest Management of the Mountainous Region, Institute of Entomology, Scientific Observing and Experimental Station of Crop Pest in Guiyang, Ministry of Agriculture, Guizhou University, Guiyang 550025, China; 4 Guizhou Plant Conservation Center, Guizhou Academy of Agriculture Science, Guiyang 550006, China

**Keywords:** *Myzus persicae*, PVY, feeding behavior, life table, nutritional component, host adaptability

## Abstract

**Simple Summary:**

Elucidating the implications of the interactions between viruses, vectors, and host plants is crucial for controlling the occurrence of viral diseases. This study evaluated the adaptability of *Myzus persicae* on PVY-infected and uninfected tobacco plants using electropenetrography and an age-stage, two-sex life table. Additionally, comparison of the amino acid and soluble sugar contents in tobacco tissue at different stages of PVY infection was carried out. The results showed that the host adaptability of *M. persicae* differed significantly according to the target plants. *M. persicae* exhibited reduced the non-probing stage and increased phloem sap ingestion on PVY-infected plants. Although the nymph development time on infected plants was significantly shorter than that on uninfected plants, *M. persicae* reared on infected plants had reduced fecundity. Our results demonstrated that PVY could alter *M. persicae*‘s adaptability by changing the nutritional quality of tobacco, with divergent effects on aphids, which were observed at different infection stages. These findings will potentially improve understanding of virus-transmission dynamics of PVY and highlight the indirect mutualistic relationship between viruses and vectors via host plants.

**Abstract:**

The indirect interaction between viruses and their insect vectors via the host plants can mediate viral transmission. Thus, elucidating these tripartite interactions is crucial for controlling the occurrence of viral diseases. This study examined the feeding behavior and life table parameters of the green peach aphid, *Myzus persicae*, using electropenetrography and an age-stage, two-sex life table on PVY-infected and uninfected tobacco plants. Furthermore, the amino acid and soluble sugar contents in tobacco tissue at different stages of PVY infection were determined. The results showed that PVY-infected plants exerted remarkable effects on the feeding activities of *M. persicae*. Aphids exhibited a reduced non-probing duration and increased phloem sap ingestion on infected plants. Although the nymph development time on the PVY-infected plants was significantly shorter than that of uninfected plants, *M. persicae* reared on infected plants had reduced fecundity and significantly shortened adult longevity. On day 12, the sugar: amino acid ratio of the PVY-infected plants was significantly higher than that of uninfected plants, whereas the opposite was observed on day 24. Our results demonstrated that PVY could alter the adaptability of *M. persicae* by modifying the nutritional quality of tobacco plants. In addition, divergent effects on aphids were observed at different infection stages, which are crucial to consider while exploring the interactions between viruses, insect vectors, and host plants. These results provided significant information for comprehending PVY spread and outbreaks.

## 1. Introduction

The majority of plant viruses are transmitted by insect vectors, with viruses potentially evolving mechanisms to alter vector activities to facilitate their spread [1]. A complex interaction between viruses, vectors, and plants exists in the natural ecosystem. Recent studies have shown that not only insect vectors can change the viral transmission, but viruses also can affect the feeding behavior and preference of insects via infected host plants [2]. Increasing evidence suggests that viruses are capable of altering the morphological, physiological, and biochemical features of host plants in ways that simultaneously influence plant interactions with vectors [3,4,5]. Moreover, the changes in plant traits caused by virus infection can indirectly affect the epidemiology of viruses [6,7].

The green peach aphid, *Myzus persicae* (Sulzer) (Hemiptera: Aphididae), is an agricultural pest that severely impacts various cultivated crops in major production areas [8]. *M. persicae* damages crops directly by feeding on the vascular bundles of plants and indirectly through the transmission of numerous viral diseases. Due to its wide host range, rapid propagation, and efficient ability to transmit plant viruses, *M. persicae* has become one of the most important pests worldwide. *Potato virus Y* (PVY) belongs to the Potyviridae family of plant viruses, which destructively reduces tobacco production and quality [9]. Prior studies have shown that PVY is effectively transmitted by *M. persicae* in a non-persistent manner, which requires sustained saliva secretion and sap ingestion by the aphids [10]. The tripartite interactions between viruses, vectors, and plants are known to crucially influence the performance of the organisms involved [11,12], as a result, the implications of the interactions between PVY, aphids, and plants in viral disease pandemics have recently attracted increased attention.

The transmission of insect-borne plant viruses depends on the feeding behavior and dispersal of their vectors to host plants [7]. Therefore, understanding vector performance on virus-infected plants is important for controlling the occurrence of viral diseases. The feeding behavior of piercing–sucking hemipteran insects can be monitored electronically using the electrical penetration graph (EPG) technique [13], which is a widely applied, reliable technique for determining the relationship between feeding activities and viral transmission to insect vectors, such as *Diaphorina citri* (Kuwayama) [14], *Bemisia tabaci* (Gennadius) [4], *M. persicae* (Sulzer) [9], and *Toxoptera citricida* (Kirkaldy) [15]. Life tables are a key qualitative assessment tool for understanding the phytophagous insect biological characteristics on plants [16]. Compared with the traditional life table, the age-stage two-sex life table construction can offer a comprehensive description of population dynamics and help illuminate the growth, development, and reproduction effects of virus-infected plants on vectors [17,18]. Virally infected plants produce toxic metabolites or change nutritional value, which can influence the host adaptability of phytophagous insects [19]. We previously demonstrated that PVY-infected tobacco plants are preferentially attractive for *M. persicae* [20]. However, the adaptability of the *M. persicae* on PVY-infected plants is yet to be investigated.

The current study evaluated the feeding behavior and life table parameters of *M. persicae* on PVY-infected and uninfected tobacco plants, to disentangle the indirect effects of the PVY on host adaptability of *M. persicae*. In addition, free amino acids and soluble sugar contents in the tobacco tissues at different stages of PVY infection were determined to explain the alteration in the adaptability of aphids. The overall study objective was to unravel the behavioral mechanisms underlying mutualistic relationship between *M. persicae* and PVY, and to understand their potential contribution to spread of PVY.

## 2. Materials and Methods

### 2.1. Test Materials

#### 2.1.1. Aphids

The *M. persicae* specimens were initially collected from a tobacco farm (29°59′ N, 106°54′ E) in September 2014, and were maintained in laboratory at the College of Plant Protection Southwest University, Chongqing, China. The aphids were reared on healthy tobacco plants (Yunyan 87) in a separate climate-controlled room at 25 ± 1 °C, 60 ± 10% relative humidity (RH), and a 16L: 8D photoperiod. Aphids were kept under the laboratory conditions for over 20 generations before all experiments.

#### 2.1.2. Virus

The tobacco veinal necrotic strain of PVY (PVY^N^) was provided by the College of Plant Protection, Shandong Agricultural University. In this study, the tobacco variety ‘Yunyan87′ was used, and plants inoculated with PVY^N^ were maintained as the virus reservoirs. To obtain PVY-infected plants, tobacco seedlings at four true-leaf stages were inoculated with PVY^N^ using the method of Shrestha et al. [21]. The three upper leaflets of tobacco plants were inoculated by mechanical inoculation. After 20 min of inoculation, the foliar abrasive carborundum on the leaves of the inoculated plants was washed gently with tap water. Control plants were mock-inoculated to mimic the effects of mechanical inoculation with the healthy tobacco tissue instead of virus sap. Plants were left to grow for 12 days, then the phenotypic changes were analyzed to determine the infection status, followed by RT-PCR validation with the primers and PCR procedures described by Liu et al. [22]. All plants were maintained inside climate chambers at 25 ± 1 °C and 60% ± 10% RH, with a photoperiod of 16:8(L:D) h.

### 2.2. Test Methods

#### 2.2.1. EPG Recording

The feeding behaviors of *M. persicae* on PVY-infected and uninfected tobacco plants were monitored using an 8-channel DC-EPG device (EPG Systems, Wageningen Agricultural University, The Netherlands). Tobacco plants inoculated with virus for 12 days were selected as the test object and heathy plants were used as the control. The experiments were performed inside a Faraday cage to avoid the effects of electrical noise in the climate chambers. Newly emerged adult apterous aphids were connected individually via their pronotum to a gold wire (15 μm diameter; 20–30 mm length) using silver conductive paint glue, then connected to the input probe of the EPG. The other electrode was placed in the soil of each potted plant. Aphids were starved for 1 h between the time of wiring and the beginning of EPG monitoring, before being placed on the abaxial side of the youngest fully expanded leaves of tobacco seedlings. The EPG parameters of *M. persicae* feeding on the two treatment tobacco plants were detected simultaneously each time, and both infected and control plants were randomly arranged in Faraday cage. The stylet activity was recorded continuously for 6 h, and 15 effective repetitions were selected for statistical analysis. Data were acquired and recorded by Stylet + for Windows software, and all recorded signals were analyzed with Probe 3.4 software (EPG Systems, Wageningen Agricultural University, The Netherlands).

#### 2.2.2. Life Table Analysis

The development, survival, and reproduction of *M. persicae* fed on PVY-infected and uninfected plants were investigated and compared. An apterous adult from the stock colonies was each transferred to the infected and uninfected plants, and after three generations of rearing, the newly emerged (0–4 h) nymphs of *M. persicae* were collected and placed individually on leaves of the respective test plants using a banister brush. A single nymph was transferred to the recently expanded leaf of one tobacco plant, and 100 nymph aphids were tested under each treatment. The experiment was conducted under the same environmental conditions as above. The survival, ecdysis, and number of newborn nymphs from each aphid were recorded at 9:00 and 21:00 every day from birth to death.

#### 2.2.3. Amino Acid and Soluble Sugar Analysis

To investigate whether PVY infection changed the tobacco nutritional quality, free amino acid and soluble sugar contents were measured in the infected and uninfected tobacco plants. The second (counted from the top) recently expanded leaves of the test plant were selected as the sample, which we observed to be preferred feeding sites for aphids in EPG assay. Plant specimens were collected 12 and 24 days after inoculation, and the corresponding uninfected plants were used as the control group. The target leaves of infected and uninfected plants were quickly cut off from the base of petiole with a blade, frozen in liquid nitrogen, and then kept at −80 °C until processing. For the analysis, 10 tobacco seedlings were randomly selected for each treatment, and the experiments were conducted in triplicates. The free amino acid content in samples were quantified using Biochrom 30 automatic analyzer (Biochrom, Cambridge, England) following the protocol of Zhang et al. [23], while anthrone colorimetric method was adopted to measure the soluble sugar content [24].

#### 2.2.4. Statistical Analysis

The *M. persicae* waveform patterns were categorized as previously described [25]. Seven distinct waveforms were identified, including np, C, potential drop (pd), F, G, E1, and E2. EPG variables were processed using the EPG-Excel Data Workbook developed by Sarria et al. [26]. The life history raw data of all aphid individuals were analyzed based on the age-stage, two-sex life table using the TWOSEX-MSChart program [18]. Accordingly, the age-stage specific survival rate (*s_xj_*), age-specific survival rate (*l_x_*), age-specific fecundity (*m_x_*), and age-specific maternity (*l_x_m_x_*) were calculated. All statistical analyses were performed in the IBM SPSS Statistics v.20.0 (SPSS, Chicago, IL, USA). Prior to analysis, normality (Shapiro–Wilk test) and homoscedasticity (Levene’s test) of variance were checked, and data that failed to fit the normal distribution were log_10_(x + 1)-transformed. Statistical comparisons of data obtained from the infected and uninfected plants were performed using one-way ANOVA with Fisher’s protected *t*-test.

## 3. Results

### 3.1. Feeding Behavior of M. Persicae on PVY-Infected and Uninfected Tobacco Plants

In the present study, we identified seven distinctly different waveforms during the *M. persicae* probing. EPG waveform patterns were categorized as previously described [8]: np, non-probing behavior; C, intercellular stylet pathway; pd, intracellular stylet puncture; F, derailed stylet mechanics; G, xylem sap ingestion; E1, phloem salivation; E2, ingestion of sieve element sap. In total, 13 non-phloem-phase parameters and 8 phloem-phase parameters were compared between infected and uninfected plants. Results showed that PVY infection of tobacco plants significantly affected the feeding behavior of *M. persicae* (Table 1).

#### 3.1.1. Non-phloem-Phase EPG Measurements

The number of probes and overall duration of np waveforms (summed across all events, then averaged per aphid) of *M. persicae* on PVY-infected plants were significantly less than those of uninfected plants (Table 1, variables 1 and 2). No significant differences were observed in the time to first probe from start of EPG, number of probes to the first E1, number of pd, mean duration of pd, total duration of C, number of F, duration of F, number of G, and duration of G between infected and uninfected plants (Table 1, variables 3, 5–7 and 9–13). The duration of the first probe on infected plants was significantly longer than that on uninfected plants, but the opposite was observed on the number of short probes (C < 3 min) (Table 1, variables 4 and 8).

#### 3.1.2. Phloem-Phase EPG Measurements

Virus infection of the plants exerted remarkable effects on the phloem-phase EPG measurements of aphids. *M. persicae* fed on PVY-infected plants showed significant reductions in the number of E1, total duration of E1, and percentage contribution of E1 to the phloem phase compared to aphids fed uninfected plants (Table 1, variables 14, 16, and 17). However, the duration of first E, number of sustained E2, and total duration of E2 were significantly greater than in the controls in aphids fed with infected plants (Table 1, variables 18 and 19). Interestingly, tobacco treatment with PVY did not significantly affect the number of E2 and the time from first probe to first E2 produced by aphids (Table 1, variables 15, 20, and 21).

### 3.2. Life Table Parameters of M. Persicae on PVY-Infected and Uninfected Tobacco Plants

The development time, adult longevity, and fecundity of *M. persicae* reared on PVY-infected and uninfected plants are presented in Table 2. The nymph developmental time and adult longevity were significantly shortened on PVY-infected plants, compared to the control. No significance difference in the adult preoviposition period (APOP) was observed, but the total preoviposition (TPOP) on infected plants was longer than that on uninfected plants. Additionally, the fecundity of *M. persicae* on PVY-infected plants was significantly decreased.

**Figure 1:**
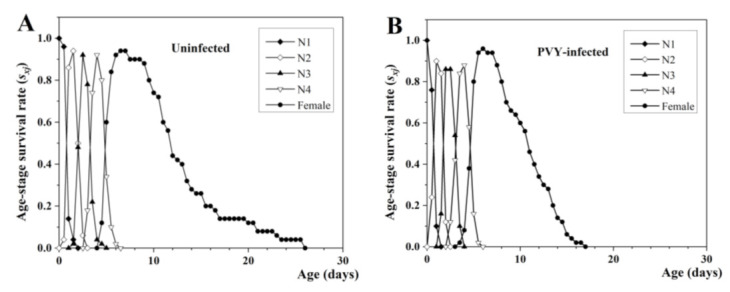
Age-stage-specific survival rate (*s_xj_*) of *M. persicae* on uninfected (**A**) and PVY-infected (**B**) tobacco plants. N1-N4 represent the 1–4 instar nymphs of *M. persicae*, respectively.

The survivorship and stage differentiation of aphids reared on two treatment tobacco plants can be estimated with the age-stage survival rate (s*_xj_*). In our study, distinct overlaps among stages were demonstrated for both infected and uninfected plants. The survivorship of larvae of *M. persicae* on uninfected plants was 94% (Figure 1A), which was lower than the 96% observed for PVY-infected plants (Figure 1B). In contrast, high mortality rates occurred in the adults fed with infected plants (Figure 1A,B).

**Figure 2:**
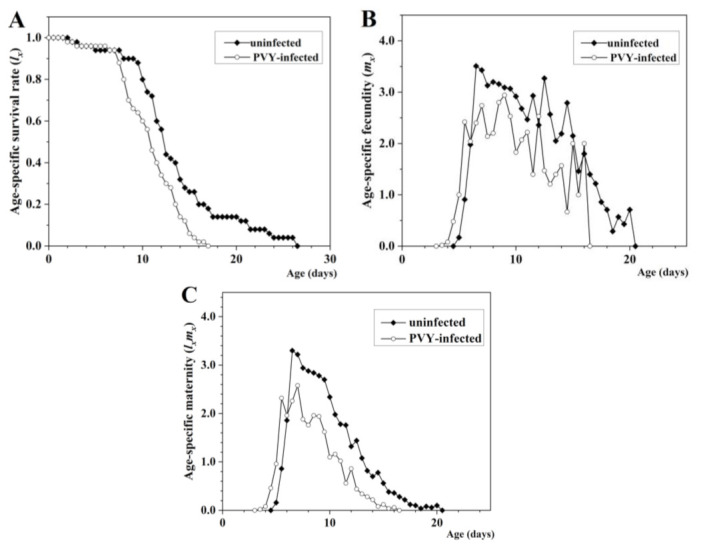
(**A**) Age-specific survival rate (*lx*), (**B**) age-specific fecundity of the total population (*m_x_*), and (**C**) age-specific maternity (*l_x_m_x_*) of *M. persicae* on uninfected and PVY-infected tobacco plants.

Figure 2 shows the age-specific survival rate (*lx*), age-specific fecundity (*m_x_*), and age-specific maternity (*l_x_m_x_*) of *M. persicae* on infected and uninfected plants. The *lx* curve describes the change in survival rate of the cohort with age, and the results show that *M. persicae* fed on PVY-infected and uninfected plants had rapid declining survivorship beginning at around day 8 (Figure 2A). For aphids fed on PVY-infected plants, the earliest occurrence of the highest fecundity peak (2.8500 offspring) was observed at the age of 7.5 d, later than the corresponding values for aphids fed on uninfected plants (3.6524 offspring, 6.5 d) (Figure 2B,C).

The effects of plants infected by PVY on the population parameters of *M. persicae* were evaluated (Table 3). The intrinsic rate of increase (*r*), finite rate of increase (*λ*), and net reproductive rate (*R_0_*) of aphids on PVY-infected plants were significantly lower than those occurring on uninfected plants. In addition, virus-infected plants had significantly shorter mean generation time (*T*) compared with the uninfected plants.

### 3.3. Quantification of Amino Acids and Soluble Sugars in PVY-Infected and Uninfected Tobacco Tissues

**Figure 3:**
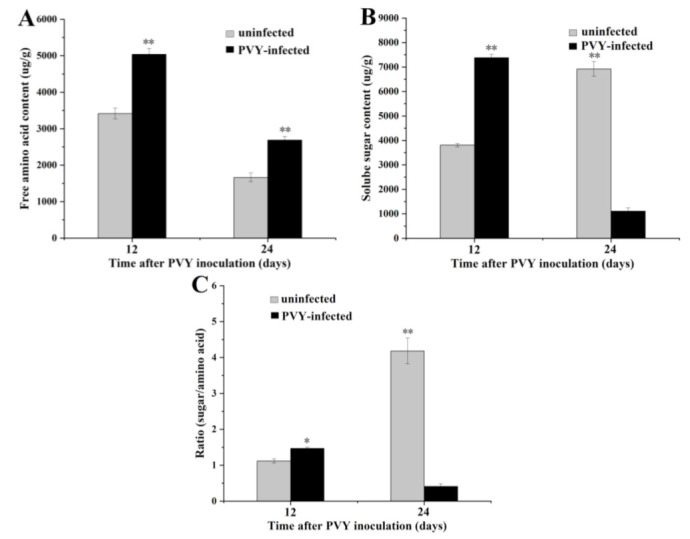
Nutrient analysis of free amino acids and soluble sugars in the leaf tissues of uninfected and PVY-infected tobacco plants. (**A**)—total free amino acid content; (**B**)—total soluble sugar content; **C**—sugar: amino acid ratio. * *p* < 0.05 and ** *p* < 0.01.

Free amino acid content in the leaf tissue of tobacco plants was enhanced at 12 and 24 days after inoculation with PVY (Figure 3A). The soluble sugar content and sugar: amino acid ratio of infected plants inoculated PVY on day 12 was significantly higher than that in the uninfected plants, whereas the opposite was observed on day 24 (Figure 3B,C).

## 4. Discussion

Plant viruses interact with their insect vectors directly or indirectly via host plants, and these tripartite interactions might promote the spread of viruses. Ingwell et al. [27] showed that infection by *Barley yellow dwarf virus* (BYDV) alters the host preferences of *Rhopalosiphum padi*, in which BYDV-infected aphids preferred to settle on healthy wheat plants. Viruses can indirectly modify the behavior and biological characteristics of insect vectors by changing the morphological, physiological, and biochemical features of host plants. For example, the better performance of *Bemisia tabaci* on tomato plants infected with *yellow leaf curl virus* (TYLCV) is likely linked to improve the host plant nutritional quality and suppress the defenses [28]. *M. persicae* transmits PVY in a non-persistent manner, and aphids can transmit the virus to another plant in seconds or minutes after being infected. Compared with persistent viruses, non-persistent viruses directly interact less closely with vector insects, which mainly indirectly affects the performance of vectors via the host plants [29]. Therefore, in the present work, we investigated the indirect effects of PVY on the adaptability of *M. persicae* by infecting the tobacco plants.

The transmission of insect-vectored diseases entails complex relationships between pathogens, vectors, and host plants [30]. The probing behavior of insect vectors during feeding on plants is associated with virus acquisition and inoculation [31]. Thus, understanding virus epidemiology requires the evaluation of feeding activities of vectors on infected plants. The feeding behaviors, particularly the superficial tissue probing and sustained phloem sap ingestion of *M. persicae*, were enhanced on plants infected by *Potato leafroll virus* (PLRV) [32]. In contrast, *Macrosiphum euphorbiae* showed delayed stylet insertion and reduced activity in the phloem vessels for infected plants [33]. Viruses can infect the host plant, and positively or negatively influence the feeding behavior of the insect vectors, which confirms the complexity in virus–vector–plant interactions. Studying the key factors and mechanisms of plant virus transmission will provide a new strategy for controlling the occurrence of viral diseases. The index of phloem factors plays a key role in evaluating the suitability of host plants for aphids [34]. This study examined the feeding behavior of *M. persicae* on infected and uninfected plants, and the results show that PVY could indirectly modify the feeding activities of *M. persicae*. Aphids feeding on infected plants exhibited reduced non-probing duration and increased phloem sap ingestion, indicating that *M. persicae* preferred to feed on PVY-infected plants. A similar result has been reported by Boquel et al. [33].

Biological characteristics such as growth, development, survival, and reproduction of phytophagous insects are greatly affected by the nutritional status of the host plant [35]. Insect-borne viruses can modify the biochemical feature of their host plants, consequently influencing the insect vectors’ host selection or biological characteristics [22,36]. A previous study revealed that insects respond to plant volatiles through a highly sensitive antennal olfactory sensilla during host searching or selection [37]. Mauck et al. [38]. showed that aphids are preferentially attracted to the elevated volatile emissions of host plants infected by *Cucumber mosaic virus* (CMV). In the present work, we used an age-stage two-sex life table to investigate the indirect effects of PVY on the biological characteristics of aphids. Notably, the effects of virus infection on aphids differed at different stages, although the development period of nymphs on PVY-infected plants was significantly shorter than that of control plants. Interestingly, aphids feeding on infected plants had reduced fecundity as well as significantly shortened adult longevity. These results demonstrated that PVY-infected plants could alter the host adaptability for *M. persicae* in a stage-dependent manner.

A recent report showed that viruses can modify the performance of vectors by changing plant nutritional quality to indirectly optimize virus transmission [39]. Currently, limited information is available on the chemical ecology of insect-vectored diseases, especially in plant pathological systems. As piercing–sucking insects, aphids use the specialized mouthparts to obtain nutrition from plants. The free amino acids and carbohydrates in plant tissues are the main nutrition for aphids [40,41]. Some studies have shown that increasing the sugar: amino acid ratio in plant tissues can improve the nutritional assimilation capacity of insects; thus, the carbon-to-nitrogen (C/N) ratio is widely recognized as a reliable indicator of plant nutritional quality [42,43,44]. In the present study, the sugar: amino acid ratio was significantly higher in PVY-infected tobacco leaf tissues on day 12 than in uninfected plants, while the opposite was observed on day 24. Our previous report also revealed that PVY-infected tobacco plants contained higher levels of free amino acids, with infection inducing the accumulation of 14 amino acids, except methionine, histidine, and proline [20].

The frequent occurrence of insect-borne plant virus diseases severely restricts yield and quality of economic crops. As a result, the tripartite interactions between viruses, aphids, and plants in viral disease pandemics have attracted considerable attention in rent years. However, numerous studies have overlooked the fact that the dynamic progression of virus infection in host plants can cause variable effects on vectors. Chen et al. [7] observed that PVY infection could enhance the nutritional quality of tobacco on 5 and 12 days after inoculation, which improved the performance of *M. persicae*, while this nutritional quality declined after 20 days, leading to early emergence and dispersal of the winged aphids. Similarly, our study showed that PVY infection disrupted the levels of carbohydrates and amino acids in leaf tissue, which modified the tobacco plant quality for aphids. Thus, PVY can indirectly alter the performance of *M. persicae* in a stage-dependent manner, which is closely associated with the nutritional status of plants at different stages of infection. Generally, viruses usually employ sophisticated strategies to overcome the distance separating host plants. Prior studies have shown that CMV can induce the production of volatile chemicals within 24 h to attract aphids in plants, but the nutritional quality of the infected plant declines after 1–2 weeks, forcing the insects to transfer to healthy plants, which facilitates virus transmission [3]. Based on our results, we speculate that PVY potentially increases the inoculation frequency by promoting the feeding behavior and shortening the nymph development time of aphids on infected plants at early infection stage, while the host plant nutritional quality for aphids decreases at a later infection stage, causing rapid aphid dispersal. This strategy may have adaptive benefits for the PVY transmission.

In conclusion, this study revealed the effects of PVY on the host adaptability of *M. persicae*. Additionally, the amino acid and soluble sugar contents in the leaf tissue of virus-infected and uninfected plants were compared. The results demonstrated that PVY can alter the *M. persicae* adaptability by changing the nutritional quality of tobacco, with divergent effects on aphids being observed at different infection stages. Our results will potentially improve the understanding of the virus-transmission dynamics of PVY and highlight the indirect mutualistic relationship between viruses and vectors via host plants. Furthermore, the novel and additional information obtained rendered better formulation of the management strategies for viral diseases.

## Figures and Tables

**Table 1:** EPG variables of *M. persicae* probing behavior on uninfected tobacco and PVY-infected tobacco during the 6 h recording period.

Variables/Per Insect	No.	EPG Parameter	Uninfected Tobacco	PVY-Infected Tobacco
Probing, pathway, and cell puncture	1	Number of probes	9.87 ± 1.60	5.27 ± 0.69 *
	2	Overall duration of np (min)	95.24 ± 19.02	46.31 ± 13.86 *
	3	Time to first probe from start of EPG (min)	16.07 ± 8.03	14.24 ± 5.14
	4	Duration of first probe (min)	27.37 ± 9.19	62.02 ± 12.59 *
	5	Number of probes to the first E1	4.33 ± 1.20	4.25 ± 0.75
	6	Number of pd	96.13 ± 12.97	91.20 ± 18.10
	7	Mean duration of pd (s)	5.37 ± 0.19	5.70 ± 0.28
	8	Number of short probes(C < 3 min)	5.87 ± 0.97	3.27 ± 0.76 *
	9	Total duration of C (min)	103.56 ± 20.34	100.20 ± 22.19
Derailed stylet mechanics	10	Number of F	0.53 ± 0.34	0.47 ± 0.13
	11	Duration of F (min)	10.50 ± 6.33	16.85 ± 6.65
Xylem ingestion	12	Number of G	0.40 ± 0.24	0.60 ± 0.38
	13	Duration of G (min)	4.04 ± 2.97	3.04 ± 1.81
Phloem salivation and ingestion	14	Number of E1	3.73 ± 0.61	2.00 ± 0.50 *
	15	Duration of first E (min)	42.24 ± 14.46	110.51 ± 27.93 *
	16	Total duration of E1 (min)	32.38 ± 6.73	15.15 ± 4.50 *
	17	Contribution of E1 to phloem phase (%)	29.84 ± 6.09	13.23 ± 4.34 *
	18	Number of E2	1.60 ± 0.38	2.00 ± 0.56
	19	Time from first probe to first E2 (min)	77.82 ± 18.06	78.07 ± 9.24
	20	Number of sustained E2(> 10 min)	0.67 ± 0.19	1.60 ± 0.39 *
	21	Total duration of E2 (min)	114.28 ± 20.87	180.47 ± 24.27 *

Data are shown as mean ± standard error. The significant differences between uninfected and PVY-infected tobacco plants are indicated with * (*p* < 0.05).

**Table 2:** Developmental time, adult longevity, APOP, TPOP, and fecundity of *M. persicae* on uninfected tobacco and PVY-infected tobacco.

Basic Statistic	Uninfected Tobacco		PVY-Infected Tobacco	
	n	Development Time (d)	n	Development Time (d)
First instar (N1)	100	1.07 ± 0.04	100	0.93 ± 0.03 *
Second instar (N2)	98	1.20 ± 0.04	98	1.05 ± 0.03 *
Third instar (N3)	96	1.27 ± 0.05	96	1.29 ± 0.04
Fourth instar (N4)	94	1.61 ± 0.05	96	1.57 ± 0.04
Preadult duration	94	5.18 ± 0.07	96	4.84 ± 0.07 *
Adult longevity	94	13.05 ± 0.72	96	10.89 ± 0.45 *
Adult preoviposition period (APOP)	94	0.58 ± 0.05	96	0.70 ± 0.06
Total preoviposition (TPOP)	94	5.31 ± 0.06	96	5.99 ± 0.10 *
Fecundity	94	42.38 ± 3.40	96	27.17 ± 2.28 *

Data are shown as mean ± SE. The significant differences between uninfected and PVY-infected tobacco plants are indicated with * (*p* < 0.05).

**Table 3:** Population parameters of *M. persicae* on uninfected tobacco and PVY-infected tobacco.

Parameter	Original		Bootstrap	
	Uninfected Tobacco	PVY-Infected Tobacco	Uninfected Tobacco	PVY-Infected Tobacco
Intrinsic rate of increase (*r*) (d^−1^)	0.4421	0.4168	0.4418 ± 0.0088	0.4164 ± 0.0090 *
Finite rate of increase (*λ*) (d^−1^)	1.5560	1.5171	1.5556 ± 0.0136	1.5166 ± 0.0136 *
Net reproductive rate (*R_0_*)	39.8400	26.0800	39.8032 ± 3.4882	26.0907 ± 2.2779 *
Mean generation time (*T*) (d)	8.3350	7.8240	8.3310 ± 0.1383	7.8240 ± 0.1787 *

Data are shown as mean ± SE. The significant differences between uninfected and PVY-infected tobacco plants are indicated with * (*p* < 0.05). The standard errors of the population parameters were calculated by using the bootstrap procedure (m = 10,000).

## Data Availability

The data presented in this study are available on request from the corresponding author.
